# Bird feathers as potential sources of pathogenic microorganisms: a new look at old diseases

**DOI:** 10.1007/s10482-018-1048-2

**Published:** 2018-02-19

**Authors:** Andrzej Miskiewicz, Paweł Kowalczyk, Sanaa Mahdi Oraibi, Krystyna Cybulska, Anna Misiewicz

**Affiliations:** 1 Department of Periodontology and Oral Diseases, Medical University of Warsaw, 18 Miodowa St., 00-246 Warsaw, Poland; 2 Department of Animal Nutrition, The Kielanowski Institute of Animal Physiology and Nutrition, Polish Academy of Sciences, Jabłonna, Poland; 3 Department of Chemistry, Microbiology and Environmental Biotechnology, Faculty of Environmental Management and Agriculture, West Pomeranian University of Technology, Słowackiego 17 Str., 71-434 Szczecin, Poland; 4 Institute of Agricultural and Food Biotechnology, Rakowiecka 36, 02-532 Warsaw, Poland

**Keywords:** Bacterial, Bird diseases, Pathogenic microorganisms, Viral and fungal serotypes

## Abstract

This article describes methods of treatment for avian zoonoses, modern antibiotic therapy and drug resistance of selected pathogens, which pose a threat to the population’s health. A tabular form has been used to present the current data from the European Union from 2011 to 2017 regarding human morbidity and mortality and the costs incurred by national health systems for the treatment of zoonoses occurring in humans and animals. Moreover, the paper includes descriptions of selected diseases, which indirectly affect birds. Scientists can obtain information regarding the occurrence of particular diseases, their aetiology, epidemiology, incubation period and symptoms caused by dangerous microorganisms and parasites. This information should be of particular interest for people who have frequent contact with birds, such as ornithologists, as well as veterinarians, farm staff, owners of accompanying animals and zoological workers. This paper presents a review used for identification and genetic characterization of bacterial strains isolated from a variety of environmental sources, e.g., bird feathers along with their practical application. We describe the bacterial, viral and fungal serotypes present on avian feathers after the slaughter process. This review also enables us to effectively identify several of the early stages of infectious diseases from heterogeneous avian research material.

## Background

Increasing morbidity from zoonoses is a significant problem in terms of global health, and they are currently considered exotic in Europe. Zoonoses are infectious or parasitic diseases transmitted by animals to humans (Spahr et al. 2018). These infections as aetiological factors can develop in humans in several ways:

- by the digestive tract, where the microorganism gets into the body via infected feed (meat) or water, which is quite often in large-scale farming. Therefore, *Escherichia* and *Salmonella* transmission to people occurs mainly through ingestion or frequent contact with infected birds. The above microorganisms can live in the external environment for a long period of time without damage to the pathogenic properties.
- by skin laceration; a break in the continuity of the skin promotes infection by pathogens of the staphylococci and streptococci families. In addition, the infection is intensified by the release of toxins into the host system.
- by the respiratory system (by inhalation of dust in which the pathogens are raised); infection occurs through direct contact of a healthy individual with contaminated excretion or through indirect contact (low hygiene at the slaughterhouse) with contaminated faeces and secretion on bird feathers (Hugh-Jones and de Vos 2002).

Humans are more susceptible to zoonoses carried by mammals than birds, and they share more diseases with them. This sharing is due to a higher degree of similarity between the intracellular environment of mammals (to which they belong), rather than birds. Many microorganisms need proper conditions and parameters to determine their host, for example, the presence of its receptor on the surface of the cells. These receptors can serve as a site for attachment and penetration into cells, which provides a pathway for the development of infection. The above situation determines the susceptibility of one animal species to a pathogen and resistance of another species. Vectors, such as insects, are also involved in the transmission of various pathogens, which may have an effect on the immune system. In the case of infecting a human with an avian zoonosis, the course of the disease is usually severe with general life-threatening symptoms. People who have been infected with an avian zoonosis require in-hospital treatment in isolation. Avian zoonoses in humans may end in death or a chronic disease requiring prolonged administration of antibiotics (Hugh-Jones and de Vos 2002).

Hence, the aim of this work is to present the aetiological factors of bird zoonoses, which are currently the most threatening to the European population. In addition, the epidemiological and economic analysis of the above infections in humans is presented. In general, zoonoses from birds can be divided by the type of the infectious agent: bacterial, viral and fungal.

## Selected bacterial zoonoses

### *Escherichia coli* serotypes

Bird feathers are the product of the epidermis that is colonized by serogroups of bacteria belonging to *E. coli* (Dho-Moulin and Fairbrother 1999). The most commonly isolated avian pathogenic *E. coli* strains are O1, O2 and O78, which are the primary cause of colicobacteriosis, inflammation of air sacs, polyserositis and bacteraemia (Mellata 2013). The factor predisposing an individual to the development of these infections is the PiCV (circovirus) infection, which affects moulting disorders but primarily damages the lymphatic tissue in the fabrician bursa, thymus, spleen and intestinal tract and leads to immunosuppression and development of secondary parenteral infections (Raue et al. 2005; Bougiouklis 2007).

Pathogenic *E. coli* are present in bird habitats, e.g., through dust and dried faeces, and may also be carried by house insects. The occurrence of colibacillosis in the flock occurs due to: keeping birds in poorly ventilated rooms, at a high density, and from malnutrition or overheating. Factors conducive to the development include immunodeficiency virus (e.g., chicken infectious anaemia virus, reo- and adenovirus infections), nutritional background (the presence of mycotoxin in the feed) and environmental factors (e.g., harmful gas admixtures: ammonia and hydrogen sulphide). The most important virulence factors of *E. coli* colonizing bird feathers are fimbriae F1 and P, aerobactin iron sequestering system and temperature-sensitive haemagglutinin (Kobayashi et al. 2010). These virulence factors promote the development of generalized infections and inhibit phagocytosis in poultry lung alveoli (in case of *E. coli* aspiration) (Stathopoulos et al. 1999). The strains IAI39 and 536 with serotypes O7:K1 and O6:K15:H31, respectively, are the aetiological agents of pyelonephritis (ExPEC). Other strains, such as Sakai, EDL933 (O157:H7 serotype), CB9615 (O55:H7 serotype), APEC01 (01 serotype) and E2348/69 (O127:H6 serotype), are responsible for causing diarrhoea with symptoms of colicobacteriosis and secondarily of *colicoseptemia* (Table 1). Among the carriers of *E. coli* strains producing *Shiga* toxin O157 (EC4115, EDL933, Sakai and TW14359, SS17, and SS52), we can distinguish the group determined as super-shedders, which is contamination of O157 ≥ 10^4^CFU/g (Ferens and Hovde 2011). In addition, due to the genomic similarity in the sequence length (5.49–5.53 Mb), G + C (50.4–50.5%) and mean length of ORF (856–898 bp) of *E. coli* strains, RFLP does not enable the identification of a specific serotype. The analysis of bacterial pathogens in breeding and wild birds permits proper clinical diagnostics in human zoonotic diseases (Table 1).

**Table 1:** Summary presentation of *E. coli* strains including serotype, strain virulence and genetic characteristics

*E. coli* strain name	Serotype	Pathotype	Genotype	Antibiotic resistance	Reference
K 12	HB101 O16	Commensal	MG1655	Kanamycin	Blattner et al. (1997), Uma et al. (2009) and Bose et al. (2017)
C2834	O2	Enterinvasive	*ipaH*+	Ampicillin, Chloramphenicol, TSX	Rosario et al. (2005)
DSM 30083^T^	O1:K1:H7	Uninary tract infections	U5/41^T^	Hydrphobic antibiotic, amingli, aminoglycosides, chloramphenicol	Meier-Kolthoff et al. (2014)
Sakai Lineage I	O157:H7	Diarrhoea	*stx2c(vh*-*b)*, *stx2a/saa/ehxA*	Β-lactams, aminoglycosides	Hayashi et al. (2001) and Brusa et al. (2017)
SS17	O157:H7	HUS	ycfR	Puromycin, acriflavine	Andrews et al. (1997) and Cote et al. (2015)
SS52	O157*nonH7*(H16, H26, H39)	pEAF	*fliC*H, *cdt, stx2*_*a*_	Β-lactams, tellurite	Katani et al. (2015) and Ferdous et al. (2016)
TW14359, Lineage II	O157, O26:H11, O103:H2	HUS	*stx1, stx2c, norV*	Β-lactams, chlorine	Batisson et al. (2003), Palaniappan et al. (2006), Deng et al. (2011) and Izac et al. (2017)
EC4115, Lineage II	O157:H7	EHEC	*gadβ*	ESBL	Zhang et al. (2007) and Winkler et al. (2015)
EDL933, Lineage I	O157:H7	EHEC	*dam, uidA, uidB,*	Carbenicillin, ampicillinstreptomycin	Foster et al. (2013) and Carone et al. (2014)

Infections with Gram-negative bacteria of birds and humans continue to be a major problem in the diagnosis, prophylaxis and treatment of humans and animals. With the introduction of large-scale poultry farming, the incidence of infections caused by *E. coli* and *Salmonella* has increased. Factors favouring this phenomenon are the intensification of poultry farming in a limited space and gaseous and microbiological pollution. Birds that are kept in difficult conditions are subjected to destructive stress, which interferes with proper immune reaction.

The presence of stress factors significantly affects the birds due to the suppression of the immune system. Therefore, in the cases of pathogen exposure, which are most commonly viruses or bacteria, there is an increased incidence in poultry. In the case of unfavourable sanitary conditions, the subjects are infected by microorganisms through

(i) the digestive tract; the most common infections are Gram-negative bacteria by ingestion of contaminated water or poultry feed. Bacteria from the genus *Salmonella* and *E. coli* can survive in the environment for long periods of time and gain virulent properties in the event of a weakened immune system of the birds.
(ii) the respiratory tract; the most common viral infections are passed by the respiratory tract (*Paramyxoviridae* and *Hepadnaviridae*) by droplet infection under conditions of excessive poultry density.
(iii) the damaged skin; infections caused by inoculation of damaged skin are usually caused by Gram-positive bacteria or by ingestion of feed containing toxins, which are usually staphylococci.

Poultry mycoplasmosis—*Mycoplasma gallisepticum* is an often diagnosed disease on poultry farms that occurs in chickens, turkeys, peacocks and pheasants. This disease induces two types of disease: sinusoidal and mixed mycoplasma infection (Mercia 2001; Hennigan et al. 2012). The most sensitive are chickens and turkeys between 6 and 10 weeks of age, especially in large breeding farms. *Mycoplasma synoviae* occurs especially in chickens and turkeys. This disease primarily causes joint infection. Birds from 2 to 20 weeks are the most sensitive to it. *Mycoplasma meleagridis* primarily occurs in turkeys and peacocks. It causes inflammation of air sacs. The most sensitive are turkeys aged 3–4 months; however, the condition also affects older birds. *Mycoplasma anatis* and *Mycoplasma anseris* are present in ducks and geese and cause disease symptoms of the respiratory system.

Clinical manifestations occur in birds from 3 weeks of age. The losses mainly relate to reproductive and commodity stock chicken and broiler chickens and turkey broilers. Infection is most often aerogenous through the respiratory system; mycoplasma penetrates the air sacs and lungs and causes inflammation (Mercia 2001; Thomas et al. 2007; Hennigan et al. 2012). From the respiratory system, the mycoplasma penetrates the bloodstream and spreads throughout the body. Mycoplasmas have an exceptional ability for parent-to-offspring transmission. The abovementioned is crucial in spreading the disease in the flock. After hatching, chickens are already infected by nets, which transmit *Mycoplasma* to the yolks. After hatching, pathogens are capable of multiplying and spreading within the hatching organism. Mycoplasmosis, if present for the first time in the flock, most often stays for an extended period of time, which is why it is a very serious problem in breeding poultry. Mycoplasma infection is often complicated with the addition to other pathogens, e.g., *E. coli, Haemophilus paragallinarum*, or viruses attacking the upper respiratory tract (Thomas et al. 2007). A complication in turkeys can be infection with infectious rhinotracheitis virus (TRT). Mycoplasma usually colonizes the surface of the mucous membranes of the respiratory and urinary tracts of various animal species. A total of 17 species of mycoplasmas have been isolated from hospitalized humans. The most important mycoplasmas causing human infections are *Mycoplasma pneumoniae* and other species from the *Mycoplasmataceae* family—*Mycoplasma hominis*, *Ureaplasma urealyticum*, and *Ureaplasma parvum*.

In European Union countries, there are programmes to eliminate the poultry infected with mycoplasmas from breeding based on serological tests. Despite the large expenditure and use of poultry flock release programmes, mycoplasmal infections have not been fully eradicated for years. However, the use of preventive vaccines significantly reduces the occurrence of disease in the flock (Thomas et al. 2007).

*Pseudotuberculosis* is a bacterial disease caused by *Yersinia pseudotuberculosis*. In birds, the disease occurs at any age, and young birds are very sensitive. Stress, worming, low hygiene, and vitamin deficiencies are also factors that predispose birds. Due to low economic value, there is only slight attention paid to the disease (Achtman et al. 1999; Robins-Browne and Hartland 2003; Lindler 2004; Carnoy et al. 2005). The sources of infection are wild and domestic birds and rodents. Infection occurs through the digestive tract from infected food and water. The pathogen can also penetrate damaged skin. The incubation period is 2–6 days. The disease is usually chronic, characterized by aversion of birds to motion, loss of appetite, diarrhoea, paralysis of the wings and lameness (Carnoy et al. 2005). Bird tuberculosis is mainly transmitted by air on small particles of mucus and may directly infect humans, as well. The aetiological agent of tuberculosis is *Mycobacterium tuberculosis*. The direct infection occurs when the individual susceptible to infection collects mycobacteria in the lungs in which they can multiply and give rise to infection. Bird treatment (high doses of tetracycline, fluoroquinolones and sulphonamides with trimethoprim) should always be combined to maintain proper hygienic conditions, rearing, and proper feeding.

*Campylobacteriosis* is a disease caused members of the genus *Campylobacter*, most commonly *Campylobacter jejuni.* These bacteria occur in the gastrointestinal tract of many animal species both domestic and wild. Studies have shown that the abovementioned bacteria play a significant role in system disorders of the digestive tract (Ryan and Ray 2004; Moore et al. 2005; Vandamme et al. 2006). An important reservoir for the disease is both breeding and wild birds, which may be entirely infected. Campylobacter infections in birds can develop as an asymptomatic or gastrointestinal tract infection. The intestinal form presents with the following symptoms: watery diarrhoea, weight loss and the worsening use of feed. In the case of *Campylobacter* infection, laying hens are recorded with reduced laying and hatching. It is worth noting that in recent years, *Campylobacter* has become more commonly known as a zoonosis than *Salmonella* (Vandamme et al. 2006). Human infection occurs through the intake of raw or undercooked infected meat, usually poultry, which leads to infections of the digestive tract, stomach and intestines. The acute, inflammatory diarrhoea most often caused by *Campylobacter jejuni* (90–95% of all infections caused by *Campylobacter* species) and *Campylobacter coli* (approximately 5% of infections) is accompanied by severe abdominal pain.

### *Salmonella* infections

In 2016, an increase in *Salmonella* infections was seen in the European Union. Bacterial genome analysis from samples collected from 260 patients infected with *Salmonella* showed two separate subspecies, which have a partially common genome sequence. Multiple locus variable-number tandem repeat (MLVA) analysis revealed two fingerprints of *Salmonella* isolates: 2-9-7-3-2 and 2-9-6-3-2. *Salmonella enteritidis* is the most commonly detected serovar, which is the aetiological factor responsible for causing non-typhoidal Salmonellosis.

Infection with the above types is most likely through contact with feathers and poultry faeces. Following the directive of the European Union (EC No. 2160/2003) regulating the conditions of poultry breeding and the sanitary conditions of the slaughterhouse, the number of infections in humans in the European Union is stable between 30,000 and 40,000. However, it is notable that two new genetically different types of Salmonella responsible for 112 (MLVA 2-9-7-3-2) and 148 (MLVA 2-9-6-3-2) infections in humans are the cause of hospitalization and death. Based on the data collected in all of the EU countries, it was observed that two types of *S. enteritidis*, MLVA 2-9-7-3-2 and 2-9-6-3-2 phage type 8, of avian origin are the aetiological factors of Salmonellosis other than typhoid fever (non-typhoidal Salmonellosis) in 6.4% of the cases of *Salmonella* infections (EFSA, ECDC 2015–2016). The Whole Genome Sequencing (WGS) analysis observed that the Enteritidis serovar is responsible for 40% of the infections.

In humans, the abovementioned serovar was responsible for over 90,000 infections throughout the European Union in 2011–2013. It is alarming that the mortality rate increased from 0.14% in 2013 to 0.24% in 2015 and almost doubled the number of hospitalizations from 7841 to 12,353 in 2015 (Table 2). Despite the constant number of non-typhoidal Salmonellosis infections, there is a growing concern regarding the increasing number of hospitalizations for *Salmonella*-induced zoonoses and the rising costs of the national health systems linked to it. The severity of infection, emerging genetic variants, and the difficulty of dealing with bird-related diseases in humans can have a significant impact on poultry breeding. Resistance to antibiotic treatment of Salmonellosis of bird origin is due to its high resistance (high multi-drug resistance (MDR)) for classic antibiotics used in the poultry industry. The MDR of the isolated bacteria among the broilers is 56%, 73% in turkeys, and the MDR in pigs is only 38% (JIACRA). The abovementioned condition results from the increasing resistance of poultry colonizing bacteria in industrial breeding, including *Salmonella* and *E. coli*, on fluoroquinolones, macrolides and tetracyclines (JIACRA and EFSA). An equally important threat to humans is the transmission of antibiotic resistance genes. Infection with Salmonella of avian origin spreads the ESBL/AmpC gene, which is especially common in poultry and is a cause of resistance to penicillin and cephalosporins. However, the plasmid mediated transmission of integrons, such as ^*bla*^CMY_2_ and ^*bla*^CTX-M_1,3,14_, observed for the first time in the US is responsible for resistance to cephamycins and monobactams (Reich et al. 2013). Therefore, the use of the above antibiotic classes in poultry farming should be avoided at this time, especially since fluoroquinolones and tetracyclines are widely used in human treatment and are the antibiotics of the most common infections in the respiratory and urinary tract. It seems optimistic that there is no relation to the use of feed containing cephalosporins of 3rd and 4th generation with antibiotic resistance among bacteria, which are the aetiological factors of bird zoonoses, especially the recently isolated MLVA subtypes 2-9-7-3-2 and MLVA 2-9-6-3-2 of *S. enteritidis.* The third generation of cephalosporins is referred to as “extremely important in the treatment of humans and animals” (Cohen et al. 2012; Liebana et al. 2013); hence, the use of cefepime and/or cefotaxime appears to be justified at present (Table 2).

**Table 2:** Incidence, hospitalization and mortality confirmed in humans for Salmonellosis and the total for all zoonoses in the European Union countries in 2013 and 2015

year	Type of zoonoses	Number of confirmed human cases	Percentage of all cases of Salmonellosis among all zoonoses	Hospitalisation reported cases	Reported deaths	Percentage of deaths due to Salmonellosis among all zoonoses	Case fatality^a^
2013	Salmonellosis	82.694	NA	7.841	59	NA	0.14
	Total no of zoonoses	313.636	26.6%	22.199	344	17.1%	0.18
2015	Salmonellosis	94.625	NA	12.353	126	NA	0.24
	Total no of zoonoses	342.651	27.6%	34.412	470	26.8%	0.23

Based on EFSA, BIOHAZ team and BIOCONTAM Unit 2013–2015 data and EU summary report on zoonoses, zoonotic agents; 2016 (10.2903/jefsa.2016.4634)

*NA* Not applicable

^a^Weighted arithmetic mean

A large number of breeding birds accumulated on a small area in a slaughterhouse promotes the intensification of the stressors. The phenomenon of stress encountered in large-scale poultry farming may have a variety of sources. This stress usually involves too many birds, poor feeding and poor maintenance (a microclimate with gaseous, particulate and microbial contamination). Birds exposed to such stressful environments are more likely to suffer from several diseases (Cohen et al. 2012; Liebana et al. 2013).

### *Bacillus* infections

Bacteria belonging to the genus *Bacillus* are Gram-positive rod-shaped and have the ability to form endospores. *Bacillus* bacteria are characterized by oxygen or relatively anaerobic metabolism and hexoses, such as glucose, are broken down into pyruvic acid (Coker et al. 2002; Lista et al. 2006). In terms of metabolic strategies, they belong to the group of chemoorganotrophs. Most bacteria of the genus *Bacillus* are recognized by the Food and Drug Administration as GRAS (generally recognized as safe); however, one of the representatives of this type of bacteria, *Bacillus anthracis,* is the aetiological agent of anthrax. The disease is one of the most crucial zoonoses not only for humans but also for carnivorous mammals. Anthrax strains are observed in water and soil, and they are transported by flies (*Muscidae*) on food. In addition, bacteria can activate even after a hundred years from spores (Maho et al. 2006; Pilo et al. 2008). People are infected with anthrax by ingesting contaminated meat or water and by skin contact with contaminated material and pulmonary anthrax by inhalation of spores. From birds, the disease is most often transmitted through direct contact with the skin and its appendages, such as feathers and claws, in large-scale poultry farms and in slaughterhouses. Anthrax still poses a danger to public health and can be a source of material loss to livestock farmers (Mohammed et al. 2002). In general, the anthrax present in birds is due to the environment of life, i.e., in free-living and breeding birds (Maho et al. 2006; Pilo et al. 2008). Free-living birds are most often infected in warm climates due to the process of sporulation, which occurs above 9–12 °C. Anthrax is most commonly found in the feathers of the Common ostrich, *Struthio camelus*, with the natural biotope the semi-deserts of Africa. Conversely, in poultry breeding, anthrax occurs primarily in the form of compact spores in chicken feed and water tanks. Anthrax has also been found in pigeon feathers; therefore, the breeding of poultry birds should be isolated from wild birds. The course of anthrax in birds is violent; the lungs have a dark colour, hyperaemia and pulmonary oedema occurs, and haemorrhagic intestinal inflammation and necrosis foci are found in the internal organs (Kruse et al. 2004). According to the current knowledge, anthrax is classified based on sequencing and DNA analysis. Presently, 89 genotypes are known, which we distinguish based on the alleles of *vrrA, vrrB*_*1*-*2*_, and *vrrC*_*1*-*2*_ genes and two loci on virulence plasmids (Keim et al. 2000; Coker et al. 2002; Lista et al. 2006; Ortatatli et al. 2012). Type A and B strains, and then the individual strains, are based on the following guidelines:

- *Single nucleotide polymorphism* (SNP) analysis to determine the phylogenetic group of bacteria,
- *Multi*-*locus VNTR analysis* (MLVA) to distinguish similar groups of partially shared sequences among the obtained isolates,
- *Single nucleotide repeat analysis* (SRA) if the above methods were not able to differentiate the tested strains (Table 3).

**Table 3:** Presentation of selected strains of Bacillus anthracis

*Bacillus anthracis* isolates		Allele size for MLVA—8 markers including virulence plasmids VNTR								Antibiotic susceptibility μg/ml			References
Strain	Type	vrrA	vrrB_1_	vrrB_2_	vrrC_1_	vrrC_2_	CG_3_	pXO_1_	pXO_2_	Amoxicillin with clavulanic acid	Fluoroquinolnes	Vancomycin	
JF3783-3788	A	314	229	162	535	604	158	135–138	139–141	≤ 2	≤ 0.25	≤1.0	Pilo et al. (2008)
Chad 1–15	Aβ	290	229	171	616	604	158	123	133	–	< 0.5	–	Mohammed et al. (2002) and Maho et al. (2006)
G_k_13,35,44	A1_a_	313	265	190	380	210	380	270	180	≤ 0.03–1	0.06–0.25	0.5–4.0	Ortatatli et al. (2012)
G_k_27	A1_b_	301											
Govi-Altai	B	10	16	7	53	17	2	8	11	–	< 0.5	–	Kruse et al. (2004) and Okutani et al. (2011)
GZBA_1_–_32_	B	2006–2011	313–349	229	581	513–542	168	132–138	143–149	0.016–0.5	0.032–0.094	0.75–5.0	Coker et al. (2002) and Okutani et al. (2011)
ASC 32	–	313	229	162	613	604	153	129	139	^a^	< 0.5	≤ 1.0	Keim et al. (2000)

Given are *variable*-*number tandem repeats (VNTR) loci vrr* -*A, B*_1–2_*C*_1–2_. CG_3_ and plasmids pX0_1–2_. together with drug susceptibility to selected antibiotics

^a^β-lactam resistance strains including ASC70 and 183; for more details see the text

Treatment and prevention against anthrax is based on the use of antibiotics: β-lactams, tetracyclines and fluoroquinolones. To date, *B. anthracis* ASC32 and ASC70 and 183 other strains (ASC32 pXO derived strains_1−/2+_) have been observed to be resistant to penicillin (Turnbull et al. 2004; Beyer and Turnbull 2009). These strains were isolated from blood infected with anthrax, fertilizers used in horticulture and feed with bone meal. If resistance is not established, the antibiotic of choice should be vancomycin. MLVA—8 analysis based on open reading frame (ORF) markers: *vrr* -*A* and *B*_1−2_*C*_1−2_. CG_3_ and plasmid DNA pX0_1−2_ in correlation with the drug susceptibility is presented in Table 3.

### Pseudomonas infections

*Pseudomonas* contains Gram-negative bacteria with a polar flagellum. Unlike the *Bacillus* genus, they do not form spores and effect the denitrification process (e.g., *Pseudomonas fluorescens*). *Pseudomonas*, in the same way as *E. coli*, metabolise hexoses via the Entner–Doudoroff pathway and obtain one molecule of ATP, NADH and NADPH as alternatives to the glycolysis pathway. Bacteria of this genus are mostly opportunistic pathogens, although we distinguish the absolute pathogens of animals and humans (*Pseudomonas aeruginosa, P. cepacia,* and *P. maltophila*). *Pseudomonas mallei* and *P.* *pseudomallei* are the aetiological factors of glanders and melioidosis. People are infected most often by inhalation and by direct contact through damaged skin causing soft tissue infections. *Pseudomonas* are usually involved in severe complications from burns and postoperative wound infections as well as respiratory tract infections. The above—mentioned complications occur in patients who are frequently hospitalized and/or hospitalized for a long period of time: especially those treated for cystic fibrosis, gastrointestinal infection, and urinary tract infections (catheters); sinusitis; ocular corneal infection; and those mechanically ventilated (Schaberg et al. 1991; Elkin and Geddes 2003; Kumaran et al. 2010).

Infections last for approximately two weeks and often result in death. Pseudomonas type virulence factors include endotoxin (LD_50_ 300 μg intravenously in mice), toxin A (ToxA) LD_50_ 0.2 μg, heat-stable haemolysin LD_50_ 5 mg, and leucocidin LD_50_ 0.4 μg. The most important factor for *Pseudomonas* virulence is toxin A (ToxA). ToxA is produced by 90% of all *Pseudomonas* strains and the mean lethal dose in mice is 0.2 μg per mouse. The toxic effect of ToxA is the inhibition of the elongation factor 2 (EF-2) by transferring the rest of the ADP-ribosyl coming from the nicotinamide adenine dinucleotide (NAD). The mechanism of action of ToxA is similar to that of the diphtheria toxin. In the case of human sepsis during infection with the ToxA strain of *Pseudomonas*, the survival chances are dramatically increased in those subjects initially exposed to *Diphtheria* producing ToxA due to the presence of the present antitoxin A antibodies (Elkin and Geddes 2003; Kumaran et al. 2010). *P. aeruginosa* together with *Aeromonas hydrophila* are the most common aetiologic factors of zoonoses of bird origin. The above bacteria, although they belong to two different taxonomic units, are characterized by common features: the biotope is the water environment (and also water at 20 °C). The above bacteria can be grown on common media (King Agar, thioglycollate), and when growing, they form β-haemolysis on the substrate with blood.

### Viral diseases

#### Marek’s disease

The most pest-prone species of poultry are primarily hens, quail and turkeys. The condition is caused by a virus belonging to the *Herpesvirus* genus. The way they penetrate the organism is through the respiratory system or gastrointestinal tract. Infection usually occurs immediately after hatching. The virus contained in the exfoliated warts of the pen still retains its virulence for more than 12 months after initiation. Infected birds show weight loss and paroxysmal symptoms. However, it often occurs that the course of this disease is very violent and no clinical symptoms were observed in humans (Ryan and Ray 2004; Koelle and Corey 2008; Johnston et al. 2011; Schiffer et al. 2014).

#### Newcastle disease

Newcastle disease is also called NCD. This disease is most often found in chickens and turkeys. Ducks and geese do not get sick but they can be carriers of the virus. The primary sources of infection are sick and dead birds (direct contact). Illness can also spread through equipment used on farms, cars, people, and wild birds (indirect contact). The infection comes through the respiratory and digestive tract and through damaged skin, conjunctiva and the mucous membrane (Csatary et al. 1999, 2000). The incubation period lasts a few days (generally 3–6 days). The virus, along with the blood, is transferred to all of the organs causing degeneration of the blood vessel walls and leading to effusion. The only protection against the dangerous virus that causes NCD is the regular vaccination of birds (Csatary et al. 1999, 2000). The disease is transmitted to humans by infected bird droppings and discharges from the beak and eyes, especially when working in poultry processing plants, among birds kept in a closure, for example, breeding chickens. Exposure of people to infected birds may cause mild conjunctivitis and flu-like symptoms, but the virus does not pose a threat to human health.

#### Infectious bronchitis

Chickens and pheasants are susceptible to infection from this disease. The disease is caused by a virus belonging to the genus Coronavirus. Initially, it was thought that the chicken disease is caused by a pathogenic, homogeneous antigenic strain, which is represented by the Massachusetts strain 41. However, the ability to create multiple variants differing from the original strain of the abovementioned led to the isolation of more than 30 serotypes and antigen variants, with the number steadily growing. Between birds, the infection spreads by the aerogenic route. The pathogen also moves between the henhouse and the farm. The incubation period is from 18 to 72 h (Cavanagh and Naqi 2003). In chickens, Infectious Bronchitis (IB) causes severe inflammatory lesions in the respiratory tract. It leads to the inflammation of the bronchial mucous membranes and the bronchial obstruction occurs as a result. As the disease progresses, the serious secretions obstruct the fork of the trachea leading to dyspnea and “breathing pumping”. In adult birds, the egg-laying capacity is rapidly reduced (within 5–7 days), and the quality of the crust is crispy and discoloured with characteristic deformities. Secondary bacterial complications should be treated with antibiotics (Cavanagh and Naqi 2003). The only effective way to avoid IB losses is through systematic and preventive vaccination. Attenuated and inactivated vaccines are used in this case. Presently, serotypes of members 4/91 are recommended in Europe to provide prototype protection and protect birds against most other antigenic IB strains (Cavanagh and Naqi 2003).

#### Avian influenza

Avian influenza is one of the most devastating diseases in chickens and turkeys, although the virus can also infect birds (pheasants, partridges, and quail), ratites (ostriches and emu), parrots and sparrows. This disease is caused by a virus of the *Orthomyxoviridae* family. The most severe form of the disease, which was originally called “Highly Pathogenic Avian Influenza (HPAI),” is included in the World Organization for Animal Health (OIE) List A contagious animal diseases. An outbreak of this disease is recorded every few years around the world. It is likely due to the occurrence of indirect or direct contact of domestic poultry with wild water birds. Infection is caused by contact with faeces of sick birds and by secretions from their eyes and nostrils. It was found that the virus also occurs in the yolk and egg white. The infected eggs do not hatch chickens, but the content in the liquid egg white is highly contagious due to the presence of virions (Panigrahy et al. 1996; Perkins and Swayne 2002). In the case of high bird density at the breeding farms, the infection can be caused by droplets from infected birds but also by such things as contaminated equipment and by means of transport. The virus incubation period ranges from several hours for 2–3 days. For official control purposes, the maximum incubation period of HPAI is 21 days.

Development of natural AI infections in poultry includes two main clinical courses: HPAI and minimally virulent bird flu (LPAI). The first includes the following symptoms: rapid fall/loss of egg production, a soft eggshell, oedema and bruising, diarrhoea, falls may be sudden with no visible symptoms, and mortality can affect up to 100% of the flock, whereas during LPAI, the following may develop: respiratory symptoms (usually mild), diarrhoea and decreased egg production in laying hens. Bird mortality is low in this case and usually does not exceed 5% although it can occur as a result of secondary infections or in young birds (occurrence of up to 97%). In the case of detection of HPAI in poultry, the strategy of action is to quickly eliminate the source of infection in the shortest possible time by introducing strict quarantine and rapid extermination of the entire poultry population in the epidemic area and destruction of the contaminated products, materials and equipment. There is also a decontamination treatment that reduces the presence of the virus by isolating the infected farms (Panigrahy et al. 1996; Perkins and Swayne 2002).

#### Infectious anaemia of chickens

Infection mainly affects young chickens. The disease causes a highly resistant virus classified as *Circovirus* and designated as CAV (Chicken Anaemia Virus) or CIA (Chicken Infectious Anaemia Virus). The main way of spreading the virus of infectious anaemia is vertically transmitted infection. In this case, the infected pullets transmit the infection to their offspring. It is also possible to spread the infection, especially in young birds, by direct contact with a patient with clinical signs and an inoculated environment (e.g., equipment and clothing) (Miller et al. 2005; Schat 2009; Quinn et al. 2015).

#### Infectious laryngotracheitis (ILT)

Chickens and pheasants are susceptible to natural infections caused by a virus belonging to the *Herpesviridae* family. ILT is one of the diseases in List B of the Animal Diseases of the World Organization for Animal Health (OIE). Infection is caused by the contact of sick birds with healthy ones. In most cases, infectious laryngotracheitis is caused by the presence of ILT virus (Fenner et al. 2015). The incubation period ranges from 4 to 12 days, and birds show signs of respiratory symptoms. The larynx and trachea are grey and easy to separate and birds often suffer from suffocation. In the typical course of ILT, the daily drop is approximately 1% (Fenner et al. 2015). Humans are not usually affected by ILT due to the thermosensitivity virus. The virions are neutralized by food thermal processing.

#### Chickenpox

Avian pox is caused by a virus in chickens, turkeys, pheasants and species of wild birds. *Avipoxvirus* belongs to the *Poxviridae* family, and like the other viruses, they significantly affect the birds. The most common cause of infection is the introduction of infected individuals into the flock. Contaminated feed and water can also be a source of infection. During the summer, mosquitoes and flies can spread infections both in the flock and between farms (Macartney and McIntyre 2008). The incubation period lasts from 4 to 20 days. Two forms of disease have been reported: dermatitis and diphtheria. The disease lasts approximately 20–30 days. Chickenpox-induced falls are very different. In the case of dermatitis, the mortality is low (1–2%) but diphtheria can reach 50% (Macartney and McIntyre 2008). In humans, the infection spreads similar to nosocomial infections. The hallmark symptom of chickenpox is a rash. Before the pox appear, there is a general malaise sensation, fever, muscle aching, loss of appetite, in some cases, a feeling of nausea. Chickenpox generally resolves within a week or two without treatment. There is no cure but a vaccine can prevent it. In the United States, the chickenpox vaccine is routinely given to children.

### Main fungal diseases

#### Aspergillosis

Aspergillosis is a group of conditions caused by *Aspergillus* fungi and most commonly by *A. fumigatus*. The disease may worsen the symptoms during other conditions in young chickens or may develop as a separate and chronic illness in the elderly. The severity of the symptoms depends on the amount of spores in the environment and the condition of the birds. The source of infection is primarily a poorly stored feed; however, the fungal spores can also be found in the mulch (mouldy straw, chips, and sawdust). Fungal respiratory infection constitutes a threat for poultry farming and comprises major cause of death in breeding birds. The disposing factor is unhygienic, dark, damp, poorly ventilated poultry houses and one-sided feeding with small amounts of vitamins (especially vitamin A) (Oglesbee 1997; Kearns 2003; Dallwig et al. 2007). The incubation period is from 1 to 10 days and depends on many factors (fungus species, route of infection, spore volumes, age and condition of the birds). Infected birds are less active, apathetic, weakened and emaciated, and they often stand with a slightly open beak. In addition, their appetite is reduced, but shortly before death it increases. In chickens with acute disease, one symptom is diarrhoea and death is preceded by the occurrence of convulsions. Paralysis of the neck may occur as well. The skin forms large and irregular scabs and often merges together. These scabs appear most often in the area of the ridge, which leads to falling of the feathers. Care should be taken to ensure the hygiene of the henhouse and the quality of the feed and litter and its proper storage. Human infection occurs by air on poultry farms from floating dust on which fungal spores occur. Inhaled from the air, fungal spores develop as pathogens in cases of underlying and severe systemic disease (e.g., tuberculosis, sarcoidosis, and AIDS). The spores penetrate the lung cavity and germinate and then form a compact biomass of mycelium. Consequently, the fungus releases toxins and allergy-causing antigens. Humans affected by the disease may show symptoms such as weight loss and chronic cough. In contrast, symptoms of extreme exhaustion occur later. Haemoptysis can occur in 50–80% of people affected by the disease (Oglesbee 1997; Kearns 2003; Dallwig et al. 2007).

#### Candidiasis

This disease is caused by *Candida* fungi. *Candida albicans* (isolated in approximately 95% of cases) is the most commonly encountered cause in poultry. Candidiasis has been reported in chickens, turkeys, guineafowls, pheasants, geese, pigeons, quail and ornamental birds such as parrots and peacocks. The infection most often occurs through contaminated litter, poor quality feed and water (James et al. 2006). The development time of the pathogen, from the time of infection to the onset of symptoms, ranges from 5 to 10 days. Symptoms associated with the disease are: reduced weight gain due to appetite suppression, deterioration in plumage quality and watery diarrhoea. Preventive measures should be based on a thorough disinfection and improved hygiene of rooms and food (James et al. 2006). There are more than 20 *Candida* species that can cause human infection, the most common of which is *C. albicans*. Symptoms of candidiasis vary depending on the area of the infected organism, and most often it develops in the mouth or throat as a result of inhaling contaminated air from enclosed farms and results in the feeling of a long-lasting dry cough.

#### Cryptococcus

*Cryptococcus* blastospores are present in the digestive tract of birds in nature. Especially high concentrations occur in the intestine of the *Columbidae* bird family (e.g., pigeons). In the case of the undermined innate immune system, the blastospores overcome through the disrupted gut barrier resulting in avian cryptococcosis. *Cryptococcus neoformans* are isolated from the faeces of pigeons or land contaminated by their faeces. Fresh larvae do not usually develop *C. neoformans* infection due to high acidic pH in the digestive tract. However, the older bird excrements are excellent nutrients in which fungi can live for up to two years. Other species do not usually get sick because the maximum fungal growth temperature is 40 °C which is lower than that in birds (Hagen et al. 2015). Mammals, including humans, are vulnerable to infection; therefore, cryptococcosis is considered a zoonosis. The infection usually occurs through direct inhalation of spores into the nasal cavity. People are most likely to be infected with *C. neoformans* by inhalation in the form of aerosol spores from dried bird faeces (Hagen et al. 2015).

## Conclusions

Transmission of the abovementioned pathogens from birds to mammals, especially humans, occasionally occurs. However, people who work professionally at bird slaughterhouses have a higher exposure, and these pathogens can be much more threatening. Nevertheless, following the outbreaks of avian influenza and *Salmonella* infections, preventive measures should be taken including transboundary infection-control strategies. The abovementioned pathogens are capable of developing serious systemic infections resulting in fatal conditions. Humans with undermined immune systems by acquired or congenital immunodeficiencies constitute a particularly vulnerable group for developing a lethal disease. Maintaining a good level of hygiene plays a fundamental role in the prevention of bird zoonoses. The occurrence of flu-like symptoms or acute food poisoning in these individuals after direct or indirect contact with birds should motivate them to seek medical attention.

## Abbreviations

AI: Avian influenza
AISS: Aerobactin iron sequestering system
Amp: Chromosomally encoded β-lactamase of class C
APEC: Avian pathogenic *Escherichia coli*
ASC: Anthrax strain collection
BIOCONTAM: EFSA unit on biological hazards and contaminants
BIOHAZ: The panel on biological hazard EFSA
CAV: Chicken anaemia virus
CIA: Chicken infection anaemia virus
Cdt: Cytolethal distending toxin
CMY: Cephalomycinase gene
CTX: Cefotaxime-hydrolysing class A β-lactamase
Dam: DNA adenine methylase
DNA: Deoxirybonucleic acid
ECDC: European Centre for Disease Prevention and Control
EHEC: Enterohemorrhagic *Escherichia coli*
ehxA: Enterohaemolysin A
EF 2: Elongation factor 2
EFSA: European Food Safety Authority
ESBL: Extended spectrum beta-lactamases
ExPEC: Extraintestinal pathogenic *Escherichia* *coli*
*fliC*H: Flagellar motor switch protein
gadβ: Glutamate decarboxylase β
GRAS: Generally recognized as safe
HPAI: Highly pathogenic avian influenza
HUS: Haemolytic-uremic syndrome
IB: Infectious bronchitis
ILT: Infectious laryngotracheitis
ipaH: Invasion plasmid antigen H
JIACRA: Joint interagency antimicrobial consumption and resistance analysis
LPAI: Low pathogenicity avian influenza
LD_50_: Median lethal dose
MDR: Multi-drug resistance
MLVA: Multiple locus variable-number tandem repeat
NADH: Nicotinamide adenine dinucleotide
NADPH: Nicotinamide adenine dinucleotide phosphate
NCD: Newcastle disease
norV: NADH:(flavo)rubredoxin reductase
OIE: World Organisation for Animal Health
ORF: Open reading frame
pEAF: *Escherichia coli* adherence factor plasmid
PiCV: Pigeon Circovirus
pXO: *Bacillus anthracis* Sterne plasmid
SNP: Single nucleotide polymorphism
SRA: Single nucleotide repeat analysis
Stx: Shiga toxin
Tox A: Toxin A
TRT: Infectious rhinotracheitis virus
UE: European Union
uidA: Beta-glucuronidase producing strains
uidB: Glucuronide carrier protein homologue
VNTR: Variable number of tandem repeats
vrrA -B, -C: Variable repeat region A, -B, -C
WGS: Whole genome sequencing
ycfR: Putative outer membrane protein
